# Can Artificial Intelligence Revolutionise Surgical Decision-Making for Appendectomy? A Narrative Review

**DOI:** 10.1177/15533506251393123

**Published:** 2025-10-29

**Authors:** Ali Murtada, Marco David Bokobza De la Rosa, Fatima Kayali, Albert Mensah, Shuaiyb Majid, Samuel N.S. Ghattas, Samuel S.S. Rezk, Ian Williams, Damian M. Bailey, Matti Jubouri, Mohamad Bashir

**Affiliations:** 1 Department of General Surgery, 97620 Glan Clwyd Hospital, Rhyl, UK; 2 Lancaster Medical School, 151569 University of Lancaster, UK; 3 Rotational Departments, University Hospital Sussex, Worthing, UK; 4 Department of General Surgery, 156680 Diana Princess of Wales Hospital, Grimsby, UK; 5 Department of Vascular Surgery, 97609 University Hospital of Wales, Cardiff, UK; 6 Neurovascular Research Laboratory, Faculty of Life Sciences and Education, 6654 University of South Wales, Pontypridd, UK; 7 12195 Hull York Medical School, University of York, UK

**Keywords:** artificial intelligence, appendicitis, appendectomy

## Abstract

**Introduction:**

Acute appendicitis is a common cause of acute abdomen in secondary care. Despite advancements in diagnostics, misdiagnosis and negative appendectomies remain significant. Artificial Intelligence (AI), particularly machine learning (ML) and deep learning, shows promise in improving diagnostic accuracy.

**Materials and Methods:**

A literature review using PubMed and Cochrane databases included studies on AI’s role in diagnosing and prognosing appendicitis. Studies relying solely on clinical or radiology reports were excluded.

**Results:**

AI models, particularly random forest (RF), logistic regression (LR), and neural networks (NN), demonstrated high diagnostic accuracy, with RF outperforming others. Machine learning methods like SVM and XGBoost (XGB) were effective in predicting appendicitis prognosis, especially in distinguishing complicated cases. AI models outperformed traditional diagnostic scores, such as the Alvarado score.

**Conclusion:**

AI has significant potential to enhance the diagnosis and prognosis of acute appendicitis, but challenges in data requirements and standardisation must be addressed for widespread clinical use.

## Introduction

Acute appendicitis is a prevalent cause of acute abdominal pain affecting both paediatric and adult populations, with an annual incidence rate of 5.7-50 patients per 100 000 individuals in developed countries.^1^ The pathophysiology of acute appendicitis arises from the obstruction of the appendiceal orifice, which can be attributed to various factors such as infections, fecaliths, lymphoid hyperplasia, or neoplasms.^2^ This condition can be categorised as simple or complex. Simple appendicitis denotes non-perforated or non-gangrenous appendicitis, with a mortality risk of 0.1% for non-gangrenous cases and 0.6% for gangrenous appendicitis. Conversely, complex appendicitis may rapidly progress to abscess or perforation, carrying an elevated mortality rate of approximately 5%.^1^

The traditional method of diagnosing appendicitis involves clinical assessment and correlating laboratory inflammatory markers. However, to enhance diagnostic accuracy, ultrasonography and computed tomography (CT) scanning are recommended.^2,3^ Nonetheless, due to the potential harm from ionising radiation used in CT scanning, it is often avoided in pregnant women and children.^4^ For these groups, ultrasonography is frequently used as an alternative. However, limitations of ultrasonography include operator dependency and patient factors. Even though the introduction of systematic preoperative imaging has reduced negative appendectomy rates in recent times, this number remains high, reaching rates of 32.8% in some studies.^3^ It is important to note, nonetheless, that although one study reported a 32.8% negative appendectomy rate,^3^ more recent analyses suggest rates have fallen below 10% in most high-resource settings, particularly with the advent of preoperative imaging and scoring systems.^1^ Additionally, 4.3% and 6.3% of children who had undergone appendectomies in the United States and Canada respectively, were found to have had unnecessary appendectomies despite a clinical and/or radiological diagnosis.^5^

Current research increasingly emphasises the application of Artificial Intelligence (AI) to support clinical decision-making and reduce the occurrence of missed and incorrect diagnoses of acute appendicitis.^6,7^ Machine learning (ML), a specific type of AI, entails training computers to learn from datasets using algorithms to make predictions or decisions. ML algorithms such as random forests (RF) and support vector machines (SVM) are utilised for classification tasks.^8^ In the case of acute appendicitis, these classification tasks can be employed to predict a diagnosis based on laboratory inflammatory marker values, patient signs and symptoms, and imaging findings. Deep learning, a subset of ML, uses multiple neural networks like convolutional neural networks (CNNs) to recognise patterns and analyse data or images, which can be valuable for interpreting CT scans in appendicitis.^9^ These forms of AI, among others, have garnered increased interest and usage in acute appendicitis diagnosis and subsequent management. This literature review will examine the effectiveness of AI in assisting clinical decision-making for this condition.

## Materials and Methods

Although this is a narrative review, a systematic process was followed to identify relevant studies. A comprehensive literature search was conducted to identify relevant studies evaluating the application of artificial intelligence (AI), including machine learning (ML) and deep learning (DL), in the diagnosis and prognosis of acute appendicitis. Searches were performed using several major electronic databases including PubMed, Ovid/MEDLINE, Google Scholar and the Cochrane Library, covering the period from January 1, 1990, to December 31, 2024. The search strategy employed a wide range of terms to capture relevant studies, including but not limited to: “acute appendicitis”, “appendicitis diagnostics”, “appendicitis imaging”, “artificial intelligence in appendicitis”, “machine learning in appendicitis”, “deep learning for appendicitis diagnosis”, “AI in diagnosis of appendicitis”, “predictive models in appendicitis”, “clinical decision support for appendicitis”, and “prognosis of appendicitis using AI”. The search focused on identifying studies that explore the role of AI, including machine learning and deep learning algorithms, in the diagnosis and prognosis of acute appendicitis.

The following Boolean search strategy was used: (“acute appendicitis” OR “appendicitis”) AND (“diagnosis” OR “prognosis”) AND (“artificial intelligence” OR “machine learning” OR “deep learning” OR “neural networks” OR “natural language processing”).

Medical Subject Headings (MeSH) were applied where appropriate. The search was limited to studies published in English and conducted on human subjects. Manual searches of reference lists from included studies and relevant reviews were also performed to ensure completeness.

Studies were eligible for inclusion if they investigated the use of AI-based tools (including ML, DL, NLP, or ensemble methods) in the diagnosis or prognosis of acute appendicitis, and reported quantitative performance metrics. The type of AI model used (eg, logistic regression, random forest, support vector machine, convolutional neural network) was noted for each study.

To assess the quality of included studies, the QUADAS-2 (Quality Assessment of Diagnostic Accuracy Studies) tool was applied to all diagnostic model studies. This tool evaluates risk of bias across four domains: patient selection, index test, reference standard, and flow/timing. Prognostic studies were evaluated based on reporting transparency, cohort description, and validation robustness. A summary table (Table 1) of QUADAS-2 assessments is included to provide an overview of the methodological rigor and potential bias of the diagnostic studies.

**Table 1.** QUADAS-2 Risk of Bias Assessment for Included Diagnostic AI Studies in Appendicitis. Evaluated Across Four Domains With Overall Bias Summarised

Study	Patient selection	Index test	Reference standard	Flow and timing	Overall risk of bias
Aydin et al. (2020)	Low	Low	Unclear	Low	Low
Hsieh et al. (2011)	Low	Low	Low	Low	Low
Gudelis et al. (2019)	Low	Low	Unclear	Low	Low
Ghareeb et al. (2022)	High	Low	Unclear	High	High
Zhao et al. (2020)	High	Unclear	Low	High	High
Park et al. (2020)	Low	Low	Unclear	Low	Low
Kim et al. (2022)	Low	Low	Low	Low	Low
Reismann et al. (2019)	Low	Low	Unclear	Low	Low
Son et al. (2012)	Low	Low	Low	Low	Low
Ting et al. (2010)	Low	Low	Low	Low	Low
Safavi et al. (2015)	Low	Low	Low	Low	Low
Prabhudesai et al. (2008)	Low	Low	Low	Low	Low
Sakai et al. (2007)	Low	Low	Low	Low	Low
Stiel et al. (2020)	Low	Low	Low	Low	Low

Given the heterogeneity of AI models, input features, and outcome measures across studies, statistical analysis was not conducted. Instead, findings were synthesized narratively with thematic emphasis on: AI model performance (diagnosis and prognosis separately); type and quality of input variables; validation approaches; study population (adult vs paediatric); and implementation potential and limitations.

## Study Quality and Validation Methods

Many included studies were conducted at single centres with small sample sizes, often under 500 participants. This raises concerns about overfitting and limited generalisability. For example, Aydin et al. trained models on 300 paediatric cases, limiting their external validity.

Only 4 studies used external validation datasets (eg, Kim et al., Reismann et al.), while most applied internal validation or cross-validation, increasing the risk of optimism bias. Additionally, paediatric studies (eg, Reismann et al., Aydin et al.) showed significantly different input features and model performance compared to adult cohorts (eg, Hsieh et al., Shahmoradi et al.), suggesting that AI models may not generalise well across age groups without retraining.

## Understanding of AI

AI encompasses the capability of computers to replicate human brain processing and functionality. AI in healthcare includes distinct subfields such as machine learning (ML), which learns patterns from numerical or image data, and natural language processing (NLP), which focuses on interpreting unstructured clinical text. While both contribute to AI decision-support systems, they operate using different data modalities and architectures.

ML, widely utilised in medical research, enables a system to improve its performance and acquire knowledge from experience by exposure to “training data” or a running algorithm. In appendicitis, ML can be applied in various ways, such as developing a diagnostic system capable of analysing diverse inputs like clinical features and laboratory results or determining the likelihood of appendicitis. Moreover, ML can be utilised to interpret medical imaging, such as ultrasound scans, to identify abnormalities, and it can even predict patient outcomes, such as post-appendectomy recovery time.^10-12^

The following passage offers an overview of various subsets of ML relevant to this review, organised into four main sections.^13^

1. Statistical and machine learning classifiers are models that categorise inputs based on known training data. Examples include logistic regression (LR), Naive Bayes, random forest (RF), support vector machine (SVM), k-nearest neighbours (KNN), and decision trees (DT).
2. Neural networks (NN) are machine learning systems inspired by the biological nervous system. They utilise interconnections to enhance processing speed and accuracy, leading to improved outcomes.
3. Ensemble learning machine models use multiple learning algorithms to achieve superior predictive performance. Examples include RF, gradient boosting (GB), extreme gradient boosting (XGB), and CatBoost.
4. Natural language processing (NLP) involves interpreting and manipulating human-generated spoken or written information, focusing on knowledge rather than just data. NLP is particularly valuable in extracting clinical information from patient records and evaluating patients’ status and outcomes.

## Role of AI in Diagnosis of Appendicitis

Various AI models differ in their effectiveness in detecting appendicitis. Research indicates that ML classification and ensemble models are taking precedence over NN, with over 30% of published data using them in the last 5 years.^10^ The most utilised individual AI models in the literature are LR, RF, DT and Artificial Neural Networks (ANN), summarised in Table 2.^7,12^

**Table 2.** Summarising Common AI Models and Their Strengths and Limitations; Artificial Neural Networks (ANN), Logistic Regression (LR), Random Forest (RF), and Decision Trees (DT)^14-16^

AI model	Description	Strengths	Limitations
ANN	ANN are the most common form of neural network. They are capable of learning complex, non-linear relationships in data through the process of forward and backward propagation	ANNs can identify complex patterns, learn hierarchical representations of features, and adapt to different data types	The abstract way in which ANNs process data complicates the interpretation of the system’s decision-making process. Additionally, ANNs require a large training dataset, which can pose limitations in studies
LR	LR serves as a statistical classifier, employing a linear model to estimate the probability of a binary outcome. In predicting diagnosis, LR utilises a logistic function to derive output probabilities, interpreting the likelihood of an event occurring	Straightforward interpretation and relatively simplistic model	Struggles with complex interactions and assumes linearity, which may not hold true in cases such as appendicitis, where age presentation is non-linear and varies among age groups.^10^
RF	RF represents a type of machine learning ensemble technique that utilises random subsets of data and features in order to construct multiple DTs that collaborate with one another	RF’s methodology helps mitigate overfitting by spreading the noise across different trees, thereby enhancing result accuracy. RF is capable of handling a large number of features and can also provide feature importance scores, thereby identifying the most relevant features	Each DT is trained independently and generates predictions based on either a majority vote or averaging of the individual tree predictions
DT	DTs function as ML classifiers, operating in a non-linear manner. They recursively divide the data into smaller groups using a series of if-else statements	DTs are easily interpretable and offer clear insights into the decision-making process. They can handle both numerical and categorical data	Due to their simplicity, they can become deep, capturing noise or patterns in the training data that do not generalise well, leading to overfitting. Additionally, DTs can be sensitive to minor changes in the training data, resulting in high variance.^11^

A study by Issayiy et al.^10^ analysed 22 articles on the diagnosis of appendicitis, examining the effectiveness of various AI models using critical performance measures. Studies that relied solely on clinician or radiology reports were not included in the analysis. The most commonly utilised AI model was LR and ANN with variable accuracy and area under the curve (AUC) reported; findings are summarised in Table 3. Issaiy et al.^10^ concluded that ANN and other NN were identified as the most effective AI models for diagnosing acute appendicitis. Most ANN studies noted limitations related to overfitting and a lack of insight into the decision-making process of the AI. The majority of the 22 studies were constrained by the data used, such as limited sample size and single-centre studies. While AUC is widely used to evaluate classifier discrimination, some studies rely only on accuracy, which may obscure model deficiencies. For instance, high accuracy in imbalanced datasets may reflect poor sensitivity. Reporting multiple metrics—sensitivity, specificity, AUC, PPV, and NPV—is essential for assessing real-world utility. However, few studies included PPV/NPV, complicating cross-study comparison. A comprehensive diagnostic AI performance metrics summary table is provided (Table 4).

**Table 3.** Summarising Findings of Issaiy et al.^10^ With Accuracy and Area Under Curve (AUC) Found for Each AI Model

AI model (number of studies)	Accuracy	AUC
LR (n = 5)	82%-87.5%	0.677-0.87
DT (n = 4)	78.7%-84.4%	0.803-0.93
RF (n = 3)	76.9%-96%	0.812-0.98
ANN (n = 5)	80%-97.8%	0.805-0.985

**Table 4.** Comprehensive Diagnostic AI Performance Metrics Summary

Study	AI model	Population	Sensitivity	Specificity	Accuracy	AUC	PPV	NPV
Aydin et al. (2020)	RF	Paediatric	97.8%	97.2%	97.5%	0.9967	Not reported	Not reported
Hsieh et al. (2011)	RF	Adult	94%	100%	96%	0.98	Not reported	Not reported
Gudelis et al. (2019)	ANN	Adult	91%	96%	93%	0.95	Not reported	Not reported
Ghareeb et al. (2022)	LR	Adult	81.2%	84.4%	87.4%	0.83	Not reported	Not reported
Zhao et al. (2020)	RF	Adult	81.2%	84.4%	83.6%	0.84	Not reported	Not reported
Park et al. (2020)	CNN	Adult	94%	90%	92%	0.94	Not reported	Not reported
Kim et al. (2022)	CNN	Paediatric	92%	95%	93%	0.93	Not reported	Not reported
Reismann et al. (2019)	ML ensemble	Paediatric	80%	87%	83%	0.9	Not reported	Not reported
Ramirez-Garcia Luna et al. (2020)	RF	Adult	91.3%	56.3%	76.9%	Not reported	Not reported	Not reported
Son et al. (2012)	DT	Adult	82.4%	78.3%	80.2%	0.803	Not reported	Not reported
Ting et al. (2010)	DT	Adult	94.5%	80.5%	78.7%	Not reported	Not reported	Not reported
Safavi et al. (2015)	ANN	Adult	97.6%	41.2%	88%	0.875	Not reported	Not reported
Prabhudesai et al. (2008)	ANN	Adult	100%	97.2%	Not reported	Not reported	Not reported	Not reported
Sakai et al. (2007)	ANN	Adult	76.7%	73.5%	Not reported	0.801	Not reported	Not reported
Akgul et al. (2021)	ANN	Paediatric	89.8%	81.2%	Not reported	0.91	Not reported	Not reported
Marcinkevics et al. (2021)	RF	Paediatric	Not reported	Not reported	Not reported	0.96	Not reported	Not reported
Stiel et al. (2020)	RF	Paediatric	87.2%	88.5%	Not reported	0.86	Not reported	Not reported
Su et al. (2022)	LR	Mixed	Not reported	Not reported	Not reported	0.84	Not reported	Not reported

A recent extensive analysis conducted by Lam et al.^7^ delved into the potential of AI in diagnosing paediatric conditions. The analysis encompassed 10 studies, with a specific focus on AI’s application in diagnosing appendicitis, and 7 of the studies addressed this area specifically. The RF algorithm was employed in 4 of the studies, while NN, DT and LR were each used in 2 studies. Some studies incorporated multiple AI methods. From the 7 studies, DT, LR, and RF were identified as the most suitable models in individual analyses, with respective AUC values of 0.93, 0.84, and 0.91. RF emerged as the most effective model in 2 studies, exhibiting AUC values ranging from 0.86 to 0.96. However, due to the small sample size, Lam et al.^7^ were unable to definitively establish the superiority of any specific AI model. Both systematic reviews concurred on the diagnostic superiority of AI over any current appendicitis score, with several studies, including Alvarado and Adult Appendicitis Score as the main comparisons.^7,10^ AI models outperformed these scores, with studies such as Park et al.^16^ and Hsieh et al.^17^ obtaining *P* values of *P* < .001 and *P* < .003 respectively.

In a study conducted by Aydin et al,^18^ six distinct AI models were trained to identify appendicitis in paediatric patients. The researchers utilised the same training set for all six models, incorporating SVM, generalised linear models, RF, DT, k-nearest neighbours, and Naive Bayes. RF consistently yielded the highest performance across all metrics: an AUC of 99.67%, accuracy of 97.45%, sensitivity of 97.79%, and specificity of 97.21%. Despite RF’s superior accuracy, the researchers opted for DT over RF due to its enhanced interpretability, enabling them to comprehend the correlation between blood variables and the ailment. This comprehension is anticipated to facilitate the development of a decision support system in the future. Even though NN appears to be the AI model most capable of creating an accurate algorithm to diagnose appendicitis, they are limited by the vast amount of training data required, as seen in Issaiy et al.^10^ RF is regarded as a better option due to its versatility in processing all the variables present in appendicitis, whether numeric or not, and due to it being less influenced by overfitting than NN. LR and DT, however, are preferable to the rest if result interpretation is necessary, because of their clear decision-making process.

## Input Variables in Diagnosis

The selection of input significantly impacts the overall performance of an AI model. When designing AI models, it’s essential to consider both the quantity and quality of the input data. Having too few input features can lead to suboptimal performance, while including too many inputs can result in overfitting the training dataset.^12^ AI models use input data to create parameters and algorithms for accurate diagnosis by giving weight to each parameter in the most favourable way. In the case of appendicitis diagnosis, older studies primarily focused on demographics and clinical variables, while newer studies included a higher percentage of laboratory and imaging-related inputs.^16,19-21^ The shift in input features away from clinical parameters is an attempt to reduce bias and move towards more standardised and less biased numerical parameters. Some studies even omitted common clinical appendicitis signs to reduce noise and subjectivity, allowing the AI to reach conclusions more efficiently.

The selection of input features for an AI model typically relies on the expertise of professionals or existing research findings. However, Xia et al.^20^ deviated from this norm by leveraging RF to identify inputs based on the mean decrease in accuracy. These selected inputs were subsequently integrated into their primary diagnostic AI model (SVM) to pinpoint crucial features and eliminate superfluous noise. The amalgamation of manual and AI-driven techniques for input selection may represent the most optimal approach for discerning the most impactful inputs.

To enhance their diagnostic capacity further, Kim et al.^11^ developed an AI model specifically designed to interpret appendiceal features from ultrasound images, significantly enhancing diagnostic accuracy. Reismann et al.^22^ further demonstrated the power of AI in image interpretation by training a model using lab results, which achieved an AUC of 0.8. When appendiceal diameter data from ultrasound scans were incorporated, the AUC increased to 0.9. Rajpurkar et al.^23^ showed that AI could interpret CT images with an AUC of 0.81 for diagnosing appendicitis. They also found that pretraining the AI on video data, rather than static images, improved diagnostic accuracy through data augmentation.

Overall, AI’s ability to interpret imaging, such as ultrasound and CT scans, has proven superior to human interpretation in identifying appendicitis, leading to more accurate diagnoses.

AI models that do not rely on imaging may still be valuable in settings where imaging is not readily available. Although developing AIs focusing on clinical signs over numerical data may pose challenges due to sample size limitations, they could still be beneficial in supporting primary care providers with limited access to costly laboratory tests.^24-26^ For instance, Shikha et al.’s^27^ AI assisted trainee doctors, enhanced their diagnostic success rate for identifying appendicitis from 70% to 97%. The AI leveraged the trainee doctors’ clinical findings in conjunction with white cell count including neutrophils to yield more accurate predictions, effectively transferring accumulated expertise from experts to trainees through their algorithm.

## Role of AI in Prognosis of Appendicitis

The prognosis for appendicitis often involves categorising it as either complicated or uncomplicated.^28^ Prompt identification of complicated appendicitis is important as these cases may require surgical intervention or interventional radiology procedures. In contrast, uncomplicated appendicitis may be considered for non-operative management in select cases.

While studies examining AI’s diagnostic capabilities are plentiful in the literature, research on AI’s prognostic abilities is less common. In contrast to appendicitis diagnosis, the most frequently used AI models in appendicitis prognosis were different. According to two separate systematic reviews by Bhandarkar et al.^12^ and Issay et al.^10^ analysing a total of 16 papers on the role of AI in appendicitis prognosis, ML models were exclusively utilised over NLP. Among the ML models, ensemble models were the most prevalent, encompassing 15 out of the 35 AI models used in the 16 studies. This is in contrast to ML classifiers, which were the second most popular, with 10 models, closely followed by statistical classifiers, accounting for 9. NNs only represented 6 AI models, with other models making up the remainder. The four most commonly used AIs within the two systematic reviews were SVM and LR each with 6 instances, and XGB/Gradient Boosting (GB) and RFs with 4 and 3 instances, respectively.

SVM are a machine learning classifier known for their exceptional ability to establish distinct boundaries between different classes, leading to improved generalisation to new data. They are frequently employed in appendicitis prediction due to their proficiency in binary classification, allowing them to identify the hyperplane that separates complex and uncomplicated cases. Nevertheless, SVMs exhibit higher sensitivity to noise when compared to other AI models like ML ensembles.^29^

XGBoost, or XGB, is a machine learning ensemble that operates similarly to Random Forests by generating multiple DTs. However, unlike RF, XGB constructs its DTs sequentially, with each tree correcting the collective prediction errors of the existing trees through gradient boosting. Although XGBoost is recognised for its exceptional performance and efficiency, it demands thorough tuning and presents challenges in interpretation due to its operational complexity. Nevertheless, its iterative predictive enhancement makes it well-suited for capturing dynamic changes in a patient’s condition.^30,31^

In the assessment of 16 papers, summarised in Table 5, LR was identified in 6 papers and emerged as the optimal model in 4 instances, thus establishing itself as the most prevalent model.^10,12^ Subsequently, XGB emerged as the most optimal model in 3 studies. While SVM was frequently used across the studies, it was deemed the most optimal AI in only 2 cases, as was RF.

**Table 5.** Studies Utilising the 4 AI Models: RF, XGB/GB, SVM & LR in Appendicitis Prognosis

	Papers about prognosis AI appendicitis	AI models appearing in papers	Ensemble ML AIs	Statistical classifiers	ML classifiers	NN
Bhandarkar et al.^12^	8	12	9 (RF n = 1)	5	2	1
			(XGB n = 2)	(LR n = 2)	(SVM n = 1)	
Issay et al.^10^	8	23	6 (RF n = 3)	4	8	5
			(GB/XGB n = 2)	(LR n = 4)	(SVM n = 5)	
Total	16	35	15	9	10	6

Comparing the accuracy of AI models across different studies proved to be challenging because of the use of various performance measures, including AUC and accuracy, with some studies not utilising either. Generally, diagnostic AIs demonstrated higher accuracy in comparison to prognostic AIs. For example, Bhandarkar et al.'s^12^ systematic review revealed an average AUC of 0.825 for diagnostic AIs, compared to 0.774 for prognostic AIs. However, some studies reported exceptionally high AUC values, such as 0.97 in Abkulut et al,^32^ highlighting the potential of AI in prognosis. The remarkable accuracy in prognostic capabilities offers numerous advantages for medical practitioners, including support in decision-making, implementation of action plans, and assessment of treatment response.

In a specific study, Li et al.^33^ identified LR as the preferred AI for distinguishing complicated and uncomplicated appendicitis cases in pregnant women. This underscores the importance of carefully selecting AI models tailored to distinct healthcare settings.

Management of complicated appendicitis often includes drainage and delayed surgery, particularly in patients with localised abscesses or poor surgical fitness. Conversely, uncomplicated appendicitis may be treated conservatively with antibiotics in selected cases. Thus, accurate classification by AI models may inform both escalation and de-escalation of care.

Numerous studies have examined postoperative complications. For instance, Eickhoff et al^34^ conducted a study that developed a RF model to forecast postoperative outcomes following perforated appendicitis. The RF model effectively predicted the necessity for intensive care with 77% accuracy, an ICU stay exceeding 24 h with 88% accuracy, complications assessed with Clavien-Dindo scores >3 with 68% accuracy, reoperation subsequent to initial appendectomy with 74% accuracy, the requirement for oral antibiotic therapy after discharge with 79% accuracy, as well as hospital stay duration and the occurrence of surgical site infections. Their AI showcased the extensive prognostic capabilities of AI, aiding in predicting whether further prophylactic treatment may be necessary. Similarly, Alramadhan et al.^34^ conducted a comparable study using ANN to forecast the risk of intra-abdominal abscess post-appendectomy with an accuracy of 89.4%.

The prognostic input characteristics were similar to those of the diagnostic AIs, but they relied more on laboratory results and had a larger number of inputs. AIs examining postoperative outcomes included unique inputs, as seen in Eickhoff et al.^34^ Their model utilised various parameters such as ASA score, type of surgery and incisions, comorbidities, blood test results, and patient demographics to predict patient outcomes.^35^

## Direction of AI in Appendicitis

This narrative review raises the question of ‘Could AI also be utilised to aid in making surgical decisions?’ AI holds significant potential in aiding the clinician’s decision making, including selecting patients for operative vs conservative management. For instance, Monsell et al.^36^ discovered that 77% of patients initially treated with antibiotics but later requiring appendectomies within 30 days exhibited acute clinical signs, underscoring the significance of further exploration into these cases using AI to potentially recognise patterns and develop an algorithm to determine whether non-surgical treatment outweighs the risks of surgery on an individual basis. Although the complexity of surgical decisions poses a challenge, the rapid advancement of technology and improvements in AI may make this a feasible prospect.

AI’s promising outcomes not only promote the benefit of improved patient safety through efficient treatment planning, recognition of deteriorating patients and reducing diagnostic error, but also potentially reduces the fiscal burdens on pressurised healthcare systems. As summarised in Table 6, literature varies regarding the preferred AI models reported, with a wide range of accuracies and AUCs reported. In the future, the widespread adoption of AI models would necessitate standardised inputs and a substantial amount of data for training.

**Table 6.** Studies Encompassing the 4 AI Models: NN, RF, LR & DT in Appendicitis Diagnosis

Authors	Year	Input	Best fit model type & performance	Performance/output
				AUC, sensitivity, specificity, accuracy
Ghareeb et al.^37^	2021	Age, gender, marital status, obesity, diabetes mellitus, hypertension, hepatitis B virus infection, hepatitis C virus infection, autoimmune diseases, pain history of similar, duration of pain, site of pain, nausea, vomiting, anorexia, body temperature, CBC, Hg, ultrasound findings	LR	Accuracy 87.4%
Zhao et al.^38^	2020	More than 800 proteins in each urine sample	RF	Accuracy 83.6%, sensitivity 81.2%, specificity 84.4
Ramirez-Garcia Luna et al.^39^	2020	Abdominal skin IRT images	RF	Accuracy 76.9%, sensitivity 91.3%, specificity 56.3%
Kang et al.^24^	2019	Rebound tenderness severity, migration, urinalysis, symptom duration, leukocytosis, neutrophil count, and CRP levels	DT	AUC 0.85
Gudelis et al.^26^	2019	Blumberg sign, pain migration, increased pain, increased pain with movement, pain when coughing, anorexia, temperature, number of leukocytes, hours of evolution, and CRP levels	ANN	AUC 0.95
Shahmoradi et al.^25^	2018	Demographic, symptoms, clinical signs, laboratory findings	LR	AUC 0.808, accuracy 83.9%, sensitivity 58.3%, specificity 93.2%
Safavi et al.^40^	2015	Age, sex, WBC, PCT, CRP, PMN	ANN	Sensitivity: 97.6%, specificity: 41.2%, accuracy: 88%, AUC: 0.875
Yoldas et al.^19^	2012	Sex, intensity of pain, relocation of pain, pain in the right lower abdominal quadrant, vomiting, temperature, guarding, bowel sounds, rebound tenderness, WBC	ANN	AUC 0.95, sensitivity 100%, specificity 97.2%
Son et al.^41^	2012	Lymphocytes, urine glucose, total bilirubin, total amylase, chloride, red blood cells, neutrophils, eosinophils, white blood cells, com- plaints, basophils, glucose, monocytes, activated partial thrombo- plastin time, urine ketone, and direct bilirubin	DT	AUC 0.803, accuracy 80.2%, sensitivity 82.4%, specificity 78.3%
Hsieh et al.^17^	2011	Age, sex, migration of pain, anorexia, nausea/vomiting, RLQ tenderness, rebounding pain, diarrhoea, progression of pain, right flank pain, body temperature, WBC, neutrophil (%), CRP, urine occult blood, haemoglobin	RF	AUC: 0.98, accuracy: 96%, sensitivity: 94% specificity: 100%
Ting et al.^42^	2010	Age, gender, migrating pain, anorexia, nausea, vomiting, RLQ tenderness, rebound pain, temperature, WBC, neutrophil count	DT	Accuracy 78.7% sensitivity: 94.5%, specificity: 80.5%
Prabhudesai et al.^43^	2008	Site of maximum pain, anorexia nausea, vomiting, site of tenderness, peritonism, temperature, WBC, neutrophil count, age, sex	ANN	Sensitivity: 100%, specificity: 97.2%
Sakai et al.^44^	2007	Gender, age, temperature, migration, tenderness at RLQ, rebound tender- ness, muscular guarding, CRP, WBC	ANN	AUC: 0.801 sensitivity: 76.7%, specificity: 73.5%
Grigull et al.^45^	2012	Age,Body temperature, blood pressure, respiratory rate, heart	ANN	
		rate, location of pain, respiration status, gastrointestinal abnormalities, presence of tumours or swellings, CNS dysfunction, skin abnormalities, presence of lymph node swelling, haemoglobin and leukocyte counts, lymphocyte and granulocyte counts, platelet, potassium, sodium, C-reactive protein level, lactate, base excess, blood glucose, urine dip-stick analysis		
Aydin et al.^18^	2020	Age, gender, white blood cell count (WBC), haemoglobin, haematocrit, red cell distribution width, mean corpuscular volume, mean corpuscular haemoglobin concentration, mean platelet volume, platelet, platelet distribution width, lymphocyte, neutrophil	DT	AUC 0.94, accuracy 94.69%, sensitivity 93.55%, specificity 96.55%
Stiel et al.^46^	2020	Age, gender, duration of abdominal pain, nausea/vomitus, stool consistency, dysuria and pyrexia, tenderness right lower quadrant, rebound tenderness, cough/hopping tenderness in the right lower quadrant, psoas sign, WBC, CRP, neutrophilia, urine analysis (nitrate, ketone and leukocytes), ultrasound findings (appendix outer diameter, surrounding tissue involvement, appendix wall hyperperfusion, free fluids, wall oedema, signs of constipation), intraoperative findings, histopathology of appendix	RF	AUC 0.86, sensitivity 87.2%, specificity 88.5%
Akgul et al.^47^	2021	Fever, tenderness at RLQ, migration of pain, vomiting, anorexia, nausea, rebound tenderness, rigidity and guarding, pain with percussion/whooping/cough, white blood cell count, absolute neutrophil count, C reactive protein, procalcitonin levels, calprotectin levels, ultrasound findings	ANN	AUC 0.91, sensitivity 89.8%, specificity 81.2%
Marcinkevics et al.^48^	2021	Age, gender, alvarado score, paediatric appendicitis score, height, weight, body mass index, peritonitis/abdominal guarding, migration of pain, tenderness in right lower quadrant, rebound tenderness, cough tenderness, psoas sign, nausea/vomiting, anorexia, body temperature, dysuria and abnormal stool, WBC, CRP, neutrophilia, urine analysis (nitrite, ketone and leukocytes), ultrasound findings (appendix outer diameter, surrounding tissue involvement, appendix wall hyper-perfusion, free fluids, wall oedema, signs of constipation)	RF	AUC 0.96
Su et al.^49^	2022	Sex, race, ethnicity, type of residence, insurance, visit year, month and day, arrival time, at least one of: Abdominal pain, constipation, diarrhoea, fever, and nausea/vomiting, initial vital signs, 5-point triage level, pain scale, 72 h revisit, injury relation, positioning, relation to medical treatment, diagnostic services (any laboratory tests or imaging tests)—Specific investigation not specified, three reasons for visiting the ED, three causes of injury recorded by the provides for each patient in the triage notes	LR	AUC 0.84

## Data Availability

The evidence used to support this review is publicly available in electronic databases including PubMed, Ovid/Medline, Scopus and Google Scholar. No new/original data was generated for the purpose of this review.*

## Conclusion

AI shows great promise for widespread implementation in healthcare, particularly in diagnosing and managing acute appendicitis. AI’s diagnostic abilities can reduce complications from late detection, optimise testing, and lower the incidence of diagnostic errors. Its use in medical imaging interpretation, such as ultrasound and CT scans, has proven superior to human interpretation, and AI models can assist healthcare providers in challenging cases where traditional methods fall short. Prognostically, AI can help identify complicated cases of appendicitis, enabling timely surgical intervention, improving postoperative outcomes, predicting complications, and helping tailor treatments to individual patient needs. While current AI models face challenges such as the requirement for large datasets and the need for standardised inputs, these hurdles are likely to be addressed as the technology continues to advance. Additionally, high-quality literature is essential to further develop our understanding of what systems are better suited for specific tasks. Despite the advancements in AI technologies, challenges remain, such as the need for large, high-quality datasets and standardised inputs.
